# Speeded Near Infrared Spectroscopy (NIRS) Response Detection

**DOI:** 10.1371/journal.pone.0015474

**Published:** 2010-11-11

**Authors:** Xu Cui, Signe Bray, Allan L. Reiss

**Affiliations:** 1 Department of Psychiatry and Behavioral Sciences, School of Medicine, Stanford University, Stanford, California, United States of America; 2 Center for Interdisciplinary Brain Sciences Research, Stanford University, Stanford, California, United States of America; The Research Center of Neurobiology-Neurophysiology of Marseille, France

## Abstract

The hemodynamic response measured by Near Infrared Spectroscopy (NIRS) is temporally delayed from the onset of the underlying neural activity. As a consequence, NIRS based brain-computer-interfaces (BCIs) and neurofeedback learning systems, may have a latency of several seconds in responding to a change in participants' behavioral or mental states, severely limiting the practical use of such systems. To explore the possibility of reducing this delay, we used a multivariate pattern classification technique (linear support vector machine, SVM) to decode the true behavioral state from the measured neural signal and systematically evaluated the performance of different feature spaces (signal history, history gradient, oxygenated or deoxygenated hemoglobin signal and spatial pattern). We found that the latency to decode a change in behavioral state can be reduced by 50% (from 4.8 s to 2.4 s), which will enhance the feasibility of NIRS for real-time applications.

## Introduction

Brain computer interface (BCI) technologies aim to interpret neural commands and translate them into communicative signals or actions, through a computer interface. These systems are particularly useful for patient populations who have lost limbs or the ability to communicate. Several technologies have been studied for recording neural activity in BCI systems, including direct electrophysiological recordings [1]–[4], electroencephalography (EEG) [5], [6], functional magnetic resonance imaging (fMRI) [7], [8] and near infrared spectroscopy (NIRS) [9]–[12]. These technologies each present their own advantages and disadvantages, and can be roughly divided according to whether they measure electrical signals, or hemodynamic signals which are an indirect measure of neural activity.

Electrophysiological recordings of neural activity have been explored for use with BCIs, and are the most direct method for measuring the spiking activity of neurons [1]–[4]. However, implanted electrodes are invasive, and developing bio-compatible devices that are stable over long periods of time is an ongoing challenge. EEG measures electrical potentials from the scalp, and has been successfully applied in several BCI applications [5], [6], but the spatial resolution of this technology is relatively poor.

FMRI has much higher spatial resolution than EEG, and several proof-of-concept studies have demonstrated that subjects can learn to issue commands using their thoughts in an MRI scanner [7], [8], [13]–[16]. With fMRI based BCI systems, participants were able to play a simple pong game [8], and navigate through a simple maze [7], by using their thoughts. However, due to the constraint that participants must lie in a scanner, fMRI is not practical for chronic BCI usage.

NIRS overcomes many of the challenges associated with the aforementioned technologies: it is non-invasive, has moderate spatial resolution and is highly portable. NIRS has been studied for use with BCIs [9]–[12]. Recently, investigators [12] demonstrated that with a NIRS based BCI, participants were able to control the movement of a toy train by performing mental arithmetic.

NIRS measures changes in local concentration of oxygenated and deoxygenated hemoglobin (oxy-Hb and deoxy-Hb) in the cerebral blood. This signal is strongly related to the BOLD signal measured with fMRI, and shows similar temporal dynamics [17]–[20]. The hemodynamic response typically peaks about 6s following stimulus onset. This delay presents an important challenge for BCI systems using hemodynamic signals: it would be prohibitively inconvenient to wait >6s following a mental command for a BCI to respond. However, for BCIs and other real-time neurofeedback applications, the raw neural signal per se is not important; what is important is how this neural signal is translated to context relevant commands or feedback. The translation algorithm plays a critical role in accurately representing or decoding the participant's true intention. While we cannot change the hemodynamic signal itself, we can in principle improve this translation algorithm to reduce the delay between the onset of mental actions, and the onset of BCI commands or feedback.

Compared to fMRI, two unique properties of NIRS make this improvement possible: higher temporal resolution and the measurement of concentration changes in both oxy- and deoxy-Hb. Typical NIRS studies measure hemodynamic changes at a rate of ∼10 Hz, thus providing a more detailed picture of the hemodynamic response than fMRI, which typically measures at 0.5–1 Hz. In principle this improved temporal resolution can be used to detect the onset of a change in brain state well before the peak of the hemodynamic response. The second advantage of NIRS is that it simultaneously measures changes in both oxy- and deoxy-Hb concentrations. This extra dimension has already been shown to be useful in motion artifact removal [20], and it might also be helpful for faster and more accurate classification of mental states.

Multivariate pattern classification techniques have been applied to BCI systems across many modalities [1], [7]. These methods are based on training a classifier to recognize which features of the data most strongly discriminate between states. Thus, these methods are useful for identifying which aspects of a signal carry the most information about the parameter of interest, in this case mental state. In the present study, we systematically explore the importance of different features of the NIRS signal to achieve the fastest and most accurate classification of mental states, using linear support vector machines (SVM). Participants in this study performed several blocks of a manual finger tapping task in order to engage a robust hemodynamic response in motor cortex. We used these data to train and test classifier performance over a range of feature spaces, including varying amounts of temporal signal history, gradient history, Hb concentrations, and spatial patterns.

## Methods

### Participants

Six healthy young adults (mean age 30.3, age range 23–37, 3 males) participated in this study. Written informed consent was obtained from all subjects, and the study protocol was approved by the Stanford University Institutional Review Board.

### Experimental procedure

We used finger tapping as an experimental task because it is known to elicit a robust hemodynamic response in motor cortex [20], [21]. The task consisted of 10s of finger tapping alternating with 20s rest periods. The first participant completed 20 periods of tapping and the remaining five participants completed 10. Before the experiment, participants were asked to sit relaxed and let their right hand rest naturally on their right knee. When the word “Tap” appeared on the screen, they began tapping the fingers on their right hand at a rate of 3–4 taps per second. When “xxx” appeared on the screen, they stopped tapping.

### NIRS data acquisition and processing

We used an ETG-4000 (Hitachi Medical, Japan) Optical Topography system to measure the concentration change of oxy-Hb and deoxy-Hb. We used the two “4×4” measurement patches provided by Hitachi. The two patches were held against participants' heads using a regular swimming cap. In each patch, 8 emitters and 8 detectors are alternatingly positioned, for a total of 32 probes, resulting in 48 measurement channels. The sampling frequency was 10Hz. The measurement area covered bilateral motor cortex, as we expected the left motor cortex to be activated by the right hand finger tapping task.

### Support Vector Machine (SVM)

We used the MatLab version of libsvm [22] (http://www.csie.ntu.edu.tw/~cjlin/libsvm/, version 2.89) to perform support vector machine classification at each time point. We compared linear and nonlinear SVM performance and found that the nonlinear SVM tended to overfit; we therefore used linear SVM in this study. In linear SVM there is one free parameter (C) which controls the penalty on the error term. In order to find the optimal value of C, we systematically investigated the performance (in terms of classification accuracy) for different values and found that C = 128 gave a stable result; we therefore used this value throughout the study.

For each participant, we trained the SVM with the first half of trials and tested on the second half. Each time point was treated as an instance, and labeled as tapping (+1) or rest (−1).

### Feature space

The main objective of this study was to find what features of the measured data are most informative for accurately classifying the mental state. Assuming the number of data points in time is N, and the number of features is m, then the feature space is of dimension N-by-m. We investigated the performance of several features:

#### Signal amplitude

Signal amplitude is simply the measured concentration change of hemoglobin. All signals were filtered using the exponential moving average (EMA) method (see below) prior to inclusion in the feature space. If only oxy-Hb is included in the feature space, the feature space is of dimension N-by-1. If both oxy- and deoxy-Hb signals are included, the feature space is N-by-2.

#### Signal history

If we include y data points from the immediate history into the feature space, then the feature space is of dimension N-by-(y+1). If both oxy- and deoxy-Hb are included, then the number of features doubles (i.e. the feature space is of dimension N-by-2 (y+1)).

**History gradient**: The gradient of the signal at time t is calculated as the difference between the signal amplitude at time t and that at time t−1, i.e. 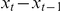 (where x_t_ is the signal amplitude at time t).

#### 2^nd^ order gradient

The second order gradient at time t is defined as 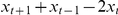, which is the gradient of the first order gradient.

#### Spatial pattern

We included the signal from multiple channels into the feature space. Channels were entered in decreasing order of the contrast-to-noise ratio (CNR, see below).

#### Full feature space

Based on the results of this investigation, the “full” feature space includes: 1s of signal amplitude history, and both oxy- and deoxy-Hb, in multiple channels (the number of channels is determined for each individual to maximize the accuracy).

### Exponential moving average (EMA)

All signals were preprocessed using an exponential moving average (EMA) [12] filter. EMA is an efficient real-time method to remove high frequency instrument noise and low frequency drift from the signal. It can be viewed as an online version of a frequency pass filtering method. EMA computes the long-term (L) and short-term (S) moving average of a time series and returns the difference between them. EMA effectively eliminates the low- and high-frequency components from the original signal with low computational cost [12]. Specifically, assume the original signal is f(t) and the filtered signal is g(t), then
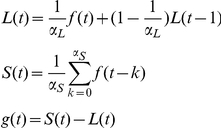
where *L(1)* = *f(1)*, α_L_ = 100 and α_S_ = 20. The sampling frequency is 10Hz and that means our short term moving average has a window of 2s.

### Contrast-to-noise-ratio (CNR)

We used CNR to quantify the signal to noise ratio (Zhang et al., 2005). Basically, CNR calculates the amplitude difference between the averaged signals during task (finger tapping) and rest, divided by the pooled standard deviation. Larger CNR indicates that the ratio of finger tapping related signal to noise is larger.
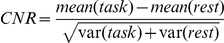
where“task” refers to the period of finger tapping, and “rest” means the rest period before finger tapping. To account for the hemodynamic delay, we chose a time window between 6–12s after the beginning of tapping as “task” and 0–5s before finger tapping as “rest”.

### Calculating delay

We calculated both onset and offset delay for each trial in the test set. The onset delay is the time between the onset of finger tapping and the first time point classified as “active”. The offset delay is the time between the offset of finger tapping, and the first time point classified as “inactive”.

## Results

We systematically evaluated the addition of features to improve classification delay, and present the results in the following order: 1) we show the result of a baseline classifier on a single participant to demonstrate that there is a long delay between the predicted and actual onset of finger tapping; 2) we test the effect of including amplitude history, and demonstrate that the classification delay can be significantly reduced; 3) we also show that including the history gradient or second order gradient does not improve the classification onset or offset delay; 4) we show that including both oxy- and deoxy-Hb improves accuracy; and 5) we find that including signals from additional channels improves accuracy and reduces delay. Finally we present the classification results using the different feature spaces for all six participants, to demonstrate that our findings generalize.

### Baseline classifier

**Figure 1:**
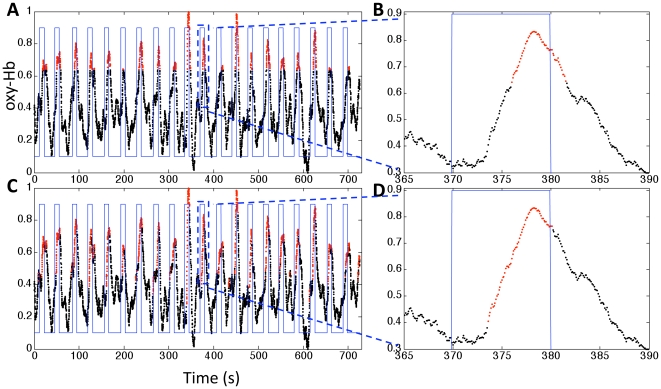
Classification results for the baseline classifier (top row) and a classifier with signal history included in the feature space (bottom row). The rectangular blue waveform indicates the onset and offset of finger tapping. A,C) The time series of oxy-Hb concentration change in channel 13 in participant 1 is plotted. The time points which are classified as active are plotted in red, and inactive in black. B,D) The time series and classification result of trial 11 is shown in more detail. The feature space of the baseline classifier is simply the amplitude of oxy-Hb in channel 13 (i.e. one dimensional), which essentially classifies based on a single threshold. It's evident from panels A and B that the classified active state (red) is delayed from the true active state (between the vertical blue lines). The onset classification delay in trial 11 is about 6s, and the offset delay is 1.6s. With 2s history of the oxy-Hb signal in channel 13 incorporated into the feature space (C and D, 21-dimensional feature space), the delay is reduced to 4s (onset) and 0.2s (offset).

We use SVM with a single feature, oxy-Hb signal in the left motor cortex, as our baseline classifier. As the feature space is one dimensional, this method simply classifies based on a single threshold. Figure 1A and B shows the classification results for participant 1 where channel 13 has the highest CNR among the measured channels, and corresponds to left motor cortex. The classified active state is delayed from the true active state by about 5s. Such a delay is a consequence of the intrinsic dynamics of the hemodynamic response. In this study, we seek to reduce this delay by using a more sophisticated classifier.

### Including signal history in the feature space

From the raw time course in Figure 1A and B we can see that if the measured signal at a certain time point is increasing, those time points are likely in the active state (finger tapping) even though the absolute signal value might not be high enough to reach the threshold. Thus, including signal history into the feature space is likely to improve classification accuracy, and reduce the delay. NIRS sampling rates are typically on the order of 10 Hz, making it possible to include fine-grained temporal information in the classifier. We incorporated 2s of signal history (20 data points) from channel 13 in participant 1 into our feature space, and used this 21 dimensional (including the signal at the current time point) feature space to classify each point of the signal. Figure 1C and D shows the classification results. Compared to Figure 1A, the classified active states are temporally more closely aligned to the true active periods. For trial 11, comparing Figure 1D to 1B, the onset delay is reduced by 2s. This demonstrates that incorporating 2s of past data points can reduce classification delay.

**Figure 2:**
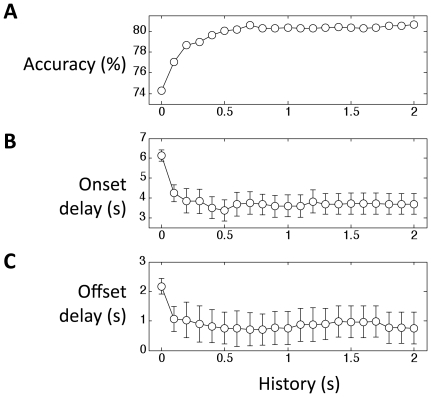
Classification accuracy, onset delay and offset delay as a function of the length of history incorporated into the feature space. A) Classification accuracy improves up to 1s, and then plateaus. B) Onset delay is reduced from 6s to 3.5s when 0.5s history is incorporated. Longer history doesn't reduce the onset delay further. C) Offset delay is reduced by 1s. Error bars indicate standard error across trials.

If we incorporate a longer history into the feature space, will the accuracy and delay reduction improve further? We investigated the effect of history length on classification performance by changing the amount of history incorporated into the feature space. Figure 2 shows the classification results (accuracy, onset delay and offset delay) for different amounts of history, in participant 1. While the inclusion of data points in the history significantly increases accuracy and reduces the delay, this improvement plateaus after 1s.

### History gradient

**Figure 3:**
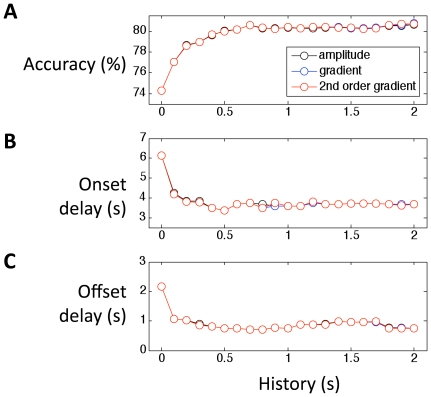
Including first and second order gradients doesn't improve classification performance. A) accuracy, B) onset delay, and C) offset delay are nearly identical with classifiers based on amplitude, and first or second order gradients.

In the previous section we used the original signal amplitude as the feature space. It is possible that, if we use the gradient (slope) in the feature space, we may further improve the performance. Here we compare three cases: using the original signal amplitude, using the gradient, and using the second order gradient. In the latter two cases (gradient and second order gradient feature spaces), we still include the amplitude at current time point as one dimension. We found the classification results are nearly identical (Figure 3) for the three cases, indicating that slope and second order gradient doesn't improve classification performance in this participant.

### oxy-Hb and deoxy-Hb

**Figure 4:**
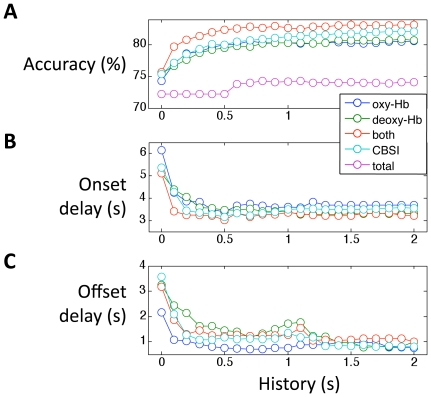
Classification results with feature spaces including oxy-Hb only (blue), deoxy-Hb only (green), both oxy-Hb and deoxy-Hb (red), CBSI corrected oxy-Hb (cyan), and total (oxy-Hb+deoxy-Hb, pink). A) accuracy, B) onset delay, and C) offset delay. Compared to the oxy-Hb only feature space, incorporating both oxy-Hb and deoxy-Hb improves the accuracy by 2.4% and reduces the onset delay by 0.3s. The total Hb signal gave the worst classification results; due to poor accuracy, we did not calculate the onset and offset delay for total-Hb.

NIRS is unique among neuroimaging technologies in that it measures both oxy-Hb and deoxy-Hb simultaneously at each location (channel). Here we investigated the feature space with different combinations of the two signals: (1) oxy-Hb only, (2) deoxy-Hb only, (3) both oxy- and deoxy-Hb, (4) correlation based signal improvement (CBSI) corrected oxy-Hb [20] , (5) total-Hb, i.e. the sum of oxy-Hb and deoxy-Hb. For each of these 5 combinations, we also investigated the performance as a function of the length of history included. Figure 4 shows the classification results using these feature spaces.

We found that oxy-Hb and deoxy-Hb perform similarly (blue and green traces) in terms of accuracy. Using both oxy-Hb and deoxy-Hb reduces the onset delay and improves the accuracy by 2.4% compared to using oxy-Hb (or deoxy-Hb) alone. Total-Hb gave the worst classification results, likely due to the negative correlation between oxy-Hb and deoxy-Hb [20] . The CBSI corrected signal performs similarly to oxy-Hb; however in data sets contaminated with motion artifact (not present in these data), CBSI may have an advantage.

### Spatial patterns

**Figure 5:**
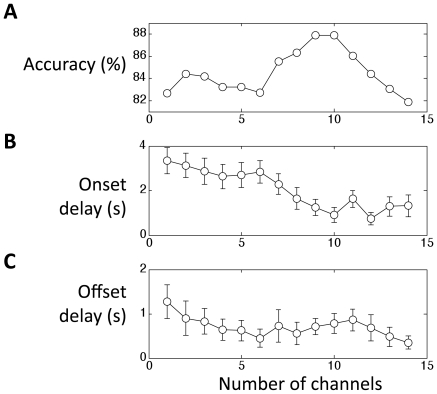
Including signals from multiple channels into the feature space improves accuracy and reduces delay in participant 1. A) accuracy, B) onset delay, and C) offset delay. We ordered all 48 channels by decreasing CNR, and then included one additional channel in each round in that order. For this participant the accuracy peaked at 9 channels and then declined due to overfitting. With 9 channels, the onset delay is reduced to 1.2s and the offset delay is reduced to 0.7s.

In the previous sections we only used the signal from the channel showing the largest CNR. In this section, we test whether the inclusion of more channels in the feature space improves performance. We first ranked all channels by CNR and then entered channels one by one into the feature space, by decreasing CNR. For each channel, we also included 1s of history (11 data points for each of oxy-Hb and deoxy-Hb). Figure 5 shows the effect of including more channels in the feature space.

### Generalization of the feature set to other participants

**Figure 6:**
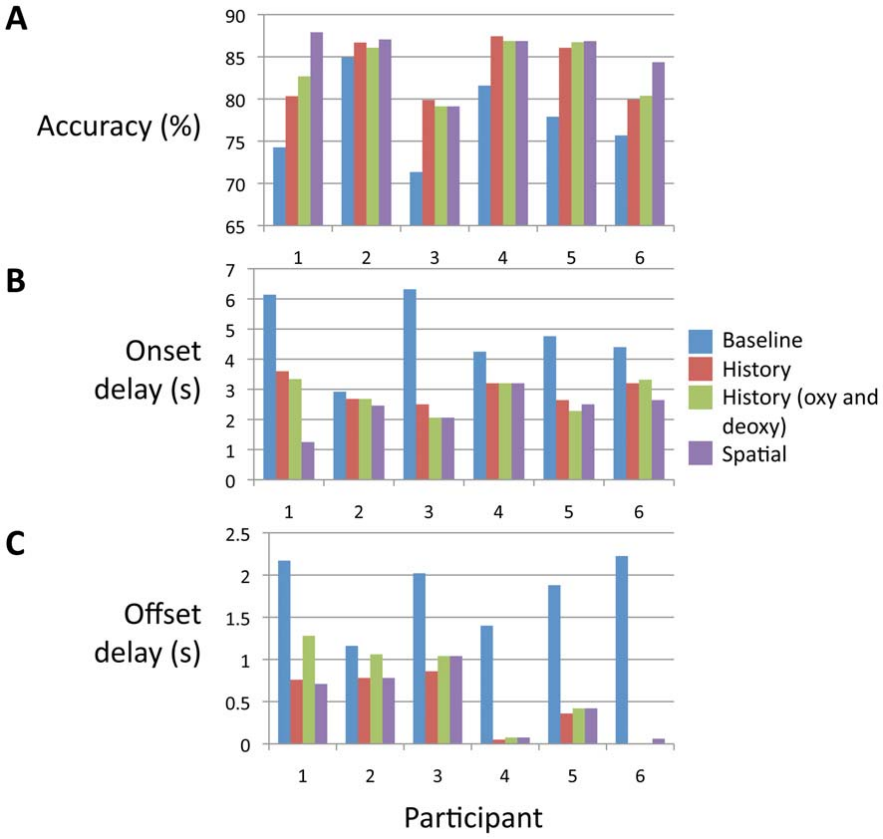
Classification results for all participants. A) accuracy, B) onset delay C) offset delay. The largest boost in accuracy and drop in delay occurs when history is included in the feature space. For some participants (#1 and #6), including deoxy-Hb or including signals from other channels also improved the performance. Comparing the classifier with the full feature space (history of oxy-Hb and deoxy-Hb, and other channels) to the baseline classifier, the average increase in accuracy is 7.7% (p = 0.004, one sample T test, degree of freedom = 5), the average reduction in onset delay is 2.4s (p = 0.02), and the average reduction in offset delay is 1.3s (p = 0.003).

We demonstrated in the previous sections that including history and multiple channels can improve the classification performance for a single participant. Here, we show that this is generally true for all participants (Figure 6). The average increase in accuracy, from the baseline classifier to the full-featured classifier, is 7.7%. The onset delay is reduced on average by 2.4s and the offset delay is reduced by 1.3s. The resultant average onset and offset delays are 2.4s and 0.5s, respectively. We performed a repeated measures ANOVA to quantify the improvement yielded by the expanded feature spaces. We found a significant effect of feature space on accuracy (F(3,15) = 17.2; p<0.005), onset delay (F(3,15) = 9.8; p<0.005), and offset delay (F(3,15) = 22.4; p<0.005).

## Discussion

In this study, we used linear SVM to classify the behavioral state of participants (finger tapping vs. rest) based on the measured NIRS signal. Specifically, we explored the usefulness of including different features, such as temporal history, temporal gradient, NIRS signals (oxy-Hb, deoxy-Hb, total-Hb and CBSI corrected signal), and spatial information, for reducing classification delay and thereby improving accuracy. We identified a specific set of features, including 1s of history information, oxy- and deoxy-Hb, and signals from multiple spatial locations, which maximally reduced the delay in a single participant. We applied the same feature space to 5 additional participants, and found that classification delays were similarly reduced across the group.

The full feature space established here was optimized for a specific participant. Although this set of features was also useful for reducing classification delays in a larger group of participants, this is by no means a one-size fits all feature space. It is also worth noting that the individual channels contributing to the feature space vary from subject to subject, depending on the CNR for each channel. Our aim here was to provide an example of the kinds of features that may be useful for reducing classification delays. Individual data sets will vary widely in terms of signal-to-noise ratio, and spatial and temporal properties. The features that provide the best classification for one data set may not be optimal for all NIRS data sets, and should ideally be optimized for individual participants.

The most important feature identified here was the signal amplitude, followed by the amplitude temporal history. For this particular data set, the points classified as ‘active’ were for the most part on an increasing slope. It is therefore quite natural that the slope would be an important feature for classifying the ‘active’ state. Including the gradient history produced similar results as including the amplitude history. This may at first seem surprising, but the gradient is just a linear function of signal at pairs of time points, and the linear classifier can also represent slope information.

NIRS measures concentration changes in both oxy- and deoxy-Hb. This paired information has already been shown to be useful for reducing motion-induced noise [20]. In two out of six participants, including both oxy- and deoxy-Hb helped to increase classification accuracy. Oxy and deoxy-Hb are typically strongly anti-correlated [20], [23]–[25], and may therefore provide some redundancy that improves classification in the presence of noise. The usefulness of including deoxy-Hb in addition to oxy-Hb may depend on the noise level, and seems to vary between individuals.

The temporal proximity of action and feedback is a key factor for associative learning [26], [27]. For example, in a rat running maze experiment, a delay of just half a second between action and reward increased the number of trials required to reach learning criteria by 5×, relative to no delay [28]. In recent years, real-time analysis of brain signals with fMRI [15], [29], and EEG [5], have made it possible to perform neurofeedback based learning studies. It has been shown that with real-time feedback of neural signals, individuals can learn to modulate regional brain activity, and reduce symptoms [15]. However, the long delay of hemodynamic responses measured by fMRI and NIRS is a potential drawback for learning efficiency. The method described here permits a shorter delay between action and neural feedback which can increase efficiency. The importance of reducing feedback delay applies equally to BCI systems; with BCIs, not only does the translation algorithm need to learn to decode the participant's true intention, the participant also needs to learn how to better control the external device. With shortened delays between mental actions and BCI response, participants should learn more quickly.

NIRS has a high sampling rate relative to the dynamics of the hemodynamic response that it measures, and it is thus arguable whether the high sampling rate of NIRS is actually useful. However, in this study we demonstrated that we can take advantage of this high sampling rate for earlier detection of brain responses. Based on our finding that the signal history is an important feature for delay reduction, it's unlikely that a similar reduction could be achieved using real-time analysis of fMRI data, due to lower temporal resolution (typically∼0.5Hz). However, fMRI has much better spatial resolution, and it's possible that the spatial information could also contribute to reducing classification delay [30].

This study demonstrates that with the judicious selection of feature space we can improve the delay to classification of a change in behavioral state. The features we have chosen here are necessarily dependent on the type of classifier we used, in this case linear SVM. The classifier extracted information about the slope that was characteristic of data points in the ‘active’ state. However, it remains to be explored whether non-linear classifiers would rely on similar feature sets.

It is also unclear whether the features chosen in this study will generalize to tasks other than finger tapping, and cortical regions other than motor cortex. For this reason we would recommend that investigators developing BCI systems should use a similar methodology to systematically choose the features that result in the most accurate classification.

To summarize, in this study we demonstrated that by including specific features, such as the history of signal amplitude, we can reduce the delay to correctly classifying a change in behavioral state using NIRS recordings. This work also emphasizes the usefulness of higher sampling rates, even for the measurement of signals that are inherently slow, and has important implications for the development of NIRS based BCIs, and neurofeedback techniques.
